# Consideration of sex as a biological variable over the history of the 5xFAD Alzheimer’s Disease mouse model

**DOI:** 10.1186/s13293-025-00788-3

**Published:** 2025-12-17

**Authors:** Julia I. Neuharth, K. Stephanie Hernandez, Jacob Bernholtz, Hadley Edwards, Adele Stewart

**Affiliations:** 1 Interdisciplinary Graduate Program in Neuroscience, University of Iowa, Iowa City, 52242 IA USA; 2 Department of Neuroscience and Pharmacology, Carver College of Medicine, University of Iowa, Iowa City, 52242 IA USA; 3 Department of Molecular Physiology and Biophysics, Carver College of Medicine, University of Iowa, Iowa City, 52242 IA USA; 4 Department of Chemistry and Biochemistry, Florida Atlantic University, Jupiter, 33458 FL USA; 5 Iowa Neuroscience Institute, University of Iowa, Iowa City, 52242 IA USA

**Keywords:** Alzheimer’s Disease, Transgenic mouse model, Sex differences, Sex as a biological variable, 5xFAD mouse

## Abstract

**Background:**

Women are nearly twice as likely to be diagnosed with Alzheimer’s Disease (AD) over their lifetime. However, historically, preclinical studies utilizing AD rodent models to define new therapeutic targets in AD treatment have neglected to consider the confounding influence of subject sex leading to a lack of mechanistic insight into the biological underpinnings of sex bias in AD.

**Methods:**

Here, we tracked choice of subject sex over the twenty-year history of the 5xFAD mouse, one of the most frequently cited pre-clinical AD models. We analyzed 1,330 primary research articles indexed on PubMed and recorded information provided regarding subject sex and/or as a rationale for not including datasets separated by sex, if noted. Trends were then plotted as a function of time ending in December 2024.

**Results:**

In the last 15 years, the number of published manuscripts on the 5xFAD model omitting information on subject sex has progressively declined. However, the proportion of studies utilizing either males only (29%) or combining data from both sexes (24%) far surpasses studies acknowledging sex as a biological variable (SABV) (< 12%) with no significant changes noted over time. On average, the ratio of male only: female only studies of 5xFAD mice hovered around 2:1. The most frequently cited reason for omitting sex-based analyses was either a lack of sex differences found (29%), accelerated development of plaque burden in 5xFAD females (17%), or the possibility of within- or between-sex variability (15%). Mention of SABV has steadily increased in studies utilizing 5xFAD mice peaking at ~ 30% of manuscripts published in 2024. However, two key confounds in the 5xFAD model, including the potential impact of an estrogen response element (ERE) and parental imprinting in the *Thy1* promoter driving transgene expression, have been largely ignored.

**Conclusions:**

The 5xFAD model represents a compelling example of how neglecting to recognize the impact of biological sex on neural function can compromise study design and data interpretation. Given sex-dependent *Thy1* promoter regulation may skew phenotypic outcomes, investigators should judiciously interpret sex differences observed in any AD mouse utilizing the *Thy1* promoter to drive transgene expression.

**Supplementary Information:**

The online version contains supplementary material available at 10.1186/s13293-025-00788-3.

## Abstract (plain language summary)

Pervasive neglect of sex as a biological variable in neuroscience research has limited identification of mechanisms driving the observed sex-bias in Alzheimer’s Disease (AD) diagnosis, age of onset, and symptomology. Here, we sought to illuminate the extent to which inattention to the modulatory influence of subject sex persists in pre-clinical studies utilizing one of the most widely distributed AD rodent models, the 5xFAD mouse. Indeed, though women are almost twice as likely to exhibit AD-related cognitive decline, we found that most research incorporating the 5xFAD line used only male subjects. Almost as prevalent were studies combining data derived from both sexes with the justification that no sex differences were found though generally no evidence was provided to support this claim. Despite efforts at the federal (circa 2016) or journal editorial (circa 2018–2020) level to encourage sex-based analyses, the last decade has seen little change in the breakdown of subject sex in reports published on 5xFAD mice. This persistent oversight in study design may contribute to inconsistent replication of datasets generated using the 5xFAD line and the failure of AD-targeted therapeutics to translate from rodent studies to efficacy in human patients. Disregard for subject sex is particularly germane to work with 5xFAD subjects as studies have shown that brain sex steroid hormone levels as well as breeding strategy are highly likely to influence transgene expression in the 5xFAD line irrespective of disease-relevant physiology.

**Supplementary Information:**

The online version contains supplementary material available at 10.1186/s13293-025-00788-3.

## Highlights

- The majority of studies utilizing the 5xFAD Alzheimer’s mouse model utilize only male subjects or combine data from both sexes.
- Whereas studies in which subject sex is not specified have decreased in the last two decades, the ratio of male-only to female-only studies of 5xFAD mice remain unchanged at a ratio around 2:1.
- The most common justifications for not considering SABV in 5xFAD model reports were a lack of sex differences found and concerns over variability between the sexes or across estrus stages in female rodents.
- Research on sex differences in 5xFAD mice have neglected to consider the confounding influence of the estrogen response element (ERE) and parental imprinting in the *Thy1* promotor driving transgene expression.

**Supplementary Information:**

The online version contains supplementary material available at 10.1186/s13293-025-00788-3.

## Introduction

Alzheimer’s Disease (AD), the leading cause of dementia worldwide, is a progressive neurodegenerative disorder characterized by key neuropathological features including the formation of aggregated amyloid beta (Aβ) plaques and neurofibrillary tangles (NFTs) consisting primarily of hyperphosphorylated tau protein [1]. Globally, an estimated 32 million individuals suffer from AD-associated dementia with an additional 69 million and 315 million persons in the prodromal and preclinical stages of the disease, respectively [2]. Women are a greater risk of developing AD (1 in 5) relative to men (1 in 10) [3] resulting in more AD diagnoses (~ 2/3) in women particularly in populations over the age of 70 [2]. Intriguingly, women with mild cognitive impairment (MCI) typically perform better than men on verbal memory tests even after controlling for hippocampal atrophy [4, 5], but later exhibit accelerated cognitive decline and loss of independence in advanced disease stages [6–10]. Psychiatric manifestations of AD also differ between the sexes with men more prone to apathy, agitation, and social dysfunction whereas women more often manifest with affective and manic symptoms [11–15]. Multiple neuroimaging studies have demonstrated accelerated brain atrophy in women relative to men [9, 16–18] and both the increased brain matter loss and accelerated cognitive decline in women may be causally linked to the higher tau burden and faster tau accumulation seen in women with AD pathology [19–23]. This sex bias in AD incidence has been attributed to several factors including the longer average lifespan of women, sex differences in contributing co-morbidities such as cardiovascular conditions and depression, and the potential influence of psychosocial and cultural factors. However, it is becoming increasingly apparent that biological and/or genetic sex modulates pathogenic mechanism(s) driving AD and may also modify the impact of underlying AD risk factors.

At the crossroads of the neural and endocrine systems, sex steroid hormones, which include progestin, estrogens, and androgens, play a critical role in brain function. Due to its significant influence on neurological structure and function, estradiol has been termed “the master regulator of the female brain” [24]. Estradiol and progesterone influence verbal memory, spatial memory tasks, fine motor skills, and fluency [25], which are behavioral domains impaired in individuals with MCI and dementia. Indeed, the sharp decline in estradiol and progesterone at menopause has been linked to an elevated risk of cognitive impairment, dementia symptoms, and AD pathology [26, 27]. In addition, ovarian resection has been shown to increase AD risk by ~ 70% in women who underwent oophorectomy prior to menopause [28, 29]. Many women of reproductive age utilize hormonal contraceptives consisting of synthetic progesterone (progestin) or a combination of progestin and synthetic estrogen [30]. Potential impacts of hormonal contraceptives on AD risk have been mixed, with most research focused on young adult women and only a few exploring the long-term effects of contraceptive use on cognitive decline in middle or older age. Some studies report no differences in cognitive performance or rates of dementia between contraceptive users and nonuser controls [31–33], while others suggest that hormonal contraceptives may enhance verbal memory [34]. Thus, it appears that maintaining steroid hormone production in brain post-menopause is critical for decreasing AD risk. Indeed, lower brain, but not circulating, estradiol was found in the samples from female AD patients relative to healthy, age-matched controls, and knockout of aromatase, the enzyme necessary for estradiol synthesis, accelerates AD-like pathology in the APP23 mouse model [35]. Interestingly, a similar impact on AD pathology was not observed following depletion of gonadal hormone production with ovariectomy [35] indicating that central, not peripheral, estrogen production is critical for neuroprotection in AD.

In addition to the potential influence of circulating steroid hormones, genetic differences between the sexes may also contribute, alone or in combination with steroid actions, to female-biased AD diagnoses. For example, homozygosity for the apolipoprotein ε4 (APOEε4) allele, the most potent genetic risk factor for idiopathic AD, is associated with more severe impairment in memory and global cognition as well as brain hypometabolism and cortical thinning in women compared to men [36, 37]. Female sex also interacts with another AD risk gene, bridging integrator 1 (BIN1), to increase disease risk in sporadic AD independent of APOE status [38]. Though many genome wide association studies (GWAS) eliminate sex chromosome-linked genes, the transcriptomic and epigenetic signature of AD also displays a pronounced sex bias with unique genetic signatures associated with risk and resilience in men vs. women with AD [39–42]. Single cell sequencing efforts have further highlighted that sex-biased gene transcription in AD may also vary across neuronal and non-neuronal cell types [43]. During embryogenesis, X-chromosome inactivation prevents overexpression of X-linked genes in females. However, some genes will escape this process leading to female-specific doubling of gene expression, a possible additional source of sex-selective disease risk. Amongst the genes known to escape X-inactivation are a handful that have been linked to amyloid accumulation or tau aggregation including *ARSD*, *DDX3X*, *FGF13*, *MAOA*, *MeCP2*, *TIMP1*, *USP9X*, and *USP11* (for review see [44]).

Despite decades of research into the mechanisms driving AD pathogenesis, the disease remains the 5th leading cause of death worldwide [45], and there are few pharmaceuticals that have proven effective in slowing cognitive decline in AD patients. Indeed, the attrition rate for AD drug candidates is particularly high. For example, between 2002 and 2012, 99.6% of AD therapies failed to progress beyond the clinical trial stage [46]. Despite the presence of detectable brain pathology, many individuals with AD will remain asymptomatic for decades prior to the emergence of clinically measurable cognitive impairment [47, 48]. Thus, it is perhaps unsurprising that there has been a shift in AD drug candidates away from amyloid and tau to parallel mechanisms driving cognitive decline [49]. Though the FDA requires clinical trials enroll, wherever possible, a gender- balanced clinical cohort, the majority of trials for leading AD treatments including the acetylcholinesterase inhibitor donepezil and NMDA antagonist memantine failed to evaluate sex differences, if any, in drug safety or efficacy [50, 51].

A key component of the drug discovery pipeline is the development and characterization of preclinical models necessary to elucidate disease drivers and screen new drugs. However, females have historically been excluded from pre-clinical studies aiming to identify the mechanisms driving neurological and neuropsychiatric disorders, with neuroscience research amongst the mostly strong skewed toward the exclusive study of male subjects [52]. The most cited justification for omission of females is concern that estrus cycling will result in endpoint variability. Though the NIH attempted to address sex bias in biomedical research with a 2016 mandate necessitating consideration of sex as a biological variable (SABV) in submitted grants, the neglect of female subjects persists [53] despite clear evidence that behavioral, morphological, physiological, and molecular traits are not inherently more variable in female rodents [54, 55]. In fact, a study surveying the top six neuroscience journals concluded that choice of subject sex is dependent on organism and the journal rather than NIH funding status [56].

To date, no studies have tracked trends in female subject inclusion in preclinical studies of AD models. We hypothesized that by evaluating choice of subject sex for a single, widely utilized and well characterized AD model over the history of the model, we might gain critical insight into when/if policy decisions have shaped choice of subjects and how subject sex selection influences experimental outcomes and interpretation with regards to disease pathogenesis and therapeutic interventions.

## Materials and methods

### Choice of 5xFAD model

**Fig. 1:**
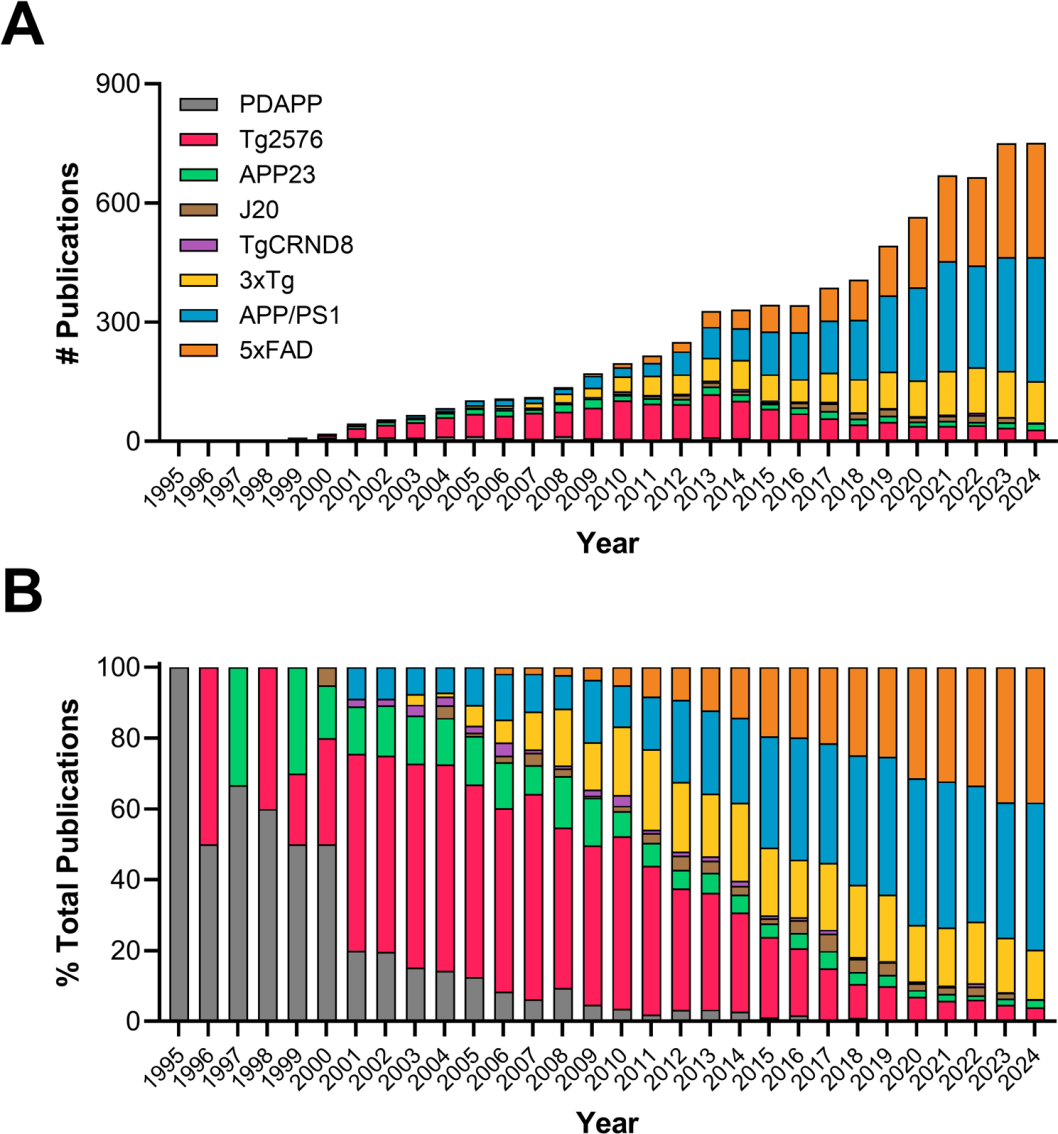
Use of AD mouse models from 1995–2024. Manuscripts available on PubMed utilizing each of the 8 most widely available genetically modified AD mouse models are provided as (**A**) total number of publications per year or (**B**) as a percent of the total AD mouse model papers published that year. Two-way ANOVA revealed a significant effect of model [F(7,158) = 15.48; *P* < 0.0001] and year [F(29,158) = 2.15; *P* = 0.0015] on the dataset in (**A**)

To select our representative AD model we first elected to limit our options to murine models, given *Mus Musculus* is the most employed model organism in neuroscience research [56]. We opted to exclude the numerous chemical insult models [57] due to a lack of standardization of dose and administration schedule across studies. We identified 8 genetically modified mouse lines expressing familial AD mutations to the amyloid precursor protein (APP), presenilin 1 (PS1) and/or tau (Table 1). When making our final model choice, we considered multiple factors including (1) length of experimental history; (2) number of available publications utilizing the model (as of January 21 st, 2025); (3) extent of current use and (4) ease of isolating research publications on the model for analysis. The utilization of AD mouse models has increased exponentially since the first models were generated in the 90s (Fig. 1A), but popularity of specific lines has shifted over time (Fig. 1A). In the last 5 years, the two most frequently cited models are the APP/PS1 and 5xFAD lines representing almost 80% of all publications (Fig. 1B). Our selection of the 5xFAD model was primarily motivated by our observation that search terms relevant to the APP/PS1 strain returned publications that predate model generation (2004) indicating a high number of irrelevant hits. To minimize labor, we ultimately settled on the 5xFAD model as our test case.

**Table 1:** Genetically modified AD mouse models

AD mouse model	Type	Promoter	Mutations	Pubmed search terms	Year generated	# of citations		
						Total	Review	Research
PDAPP	Transgenic	*PDGF*	APP-V717F	PDAPP	1995	166	12	154
Tg2576	Transgenic	*PrP*	APP695 (K670N, M671L)	tg2576	1996	1222	35	1187
APP23	Transgenic	*Thy1.2*	APP751 (K670N, M671L)	app23	1997	296	15	281
J20	Transgenic	*PDGF-*β	APP695 (K670N, M671L, V717F)	j20 ad	2000	151	5	146
TgCRND8	Transgenic	*PrP*	APP695 (K670N, M671L, V717F)	crnd8	2001	57	1	56
3xTg	Transgenic/ Knock-In	*Thy1.2*	APP (K670N, M671L), PS1 (M146V), Tau (P301L)	3xtg ad	2003	1108	37	1071
APP/PS1	Transgenic	*PrP*	APP695 (K670N, M671L), PS1 (ΔE9)	“app/ps1 mice”	2004	2664	30	2634
5xFAD	Transgenic	*Thy1*	APP695 (K670N, M671L, I716V, V717I), PS1 (M146L, L286V)	5xfad	2006	1558	21	1537

Summary data on the 8 most common genetically modified AD mouse models. Included are the PubMed search terms used to isolate model-relevant publications including the number classified by PubMed as reviews, clinical trials, or book chapters

### Inclusion criteria, manuscript coding, and data collection 

**Fig. 2:**
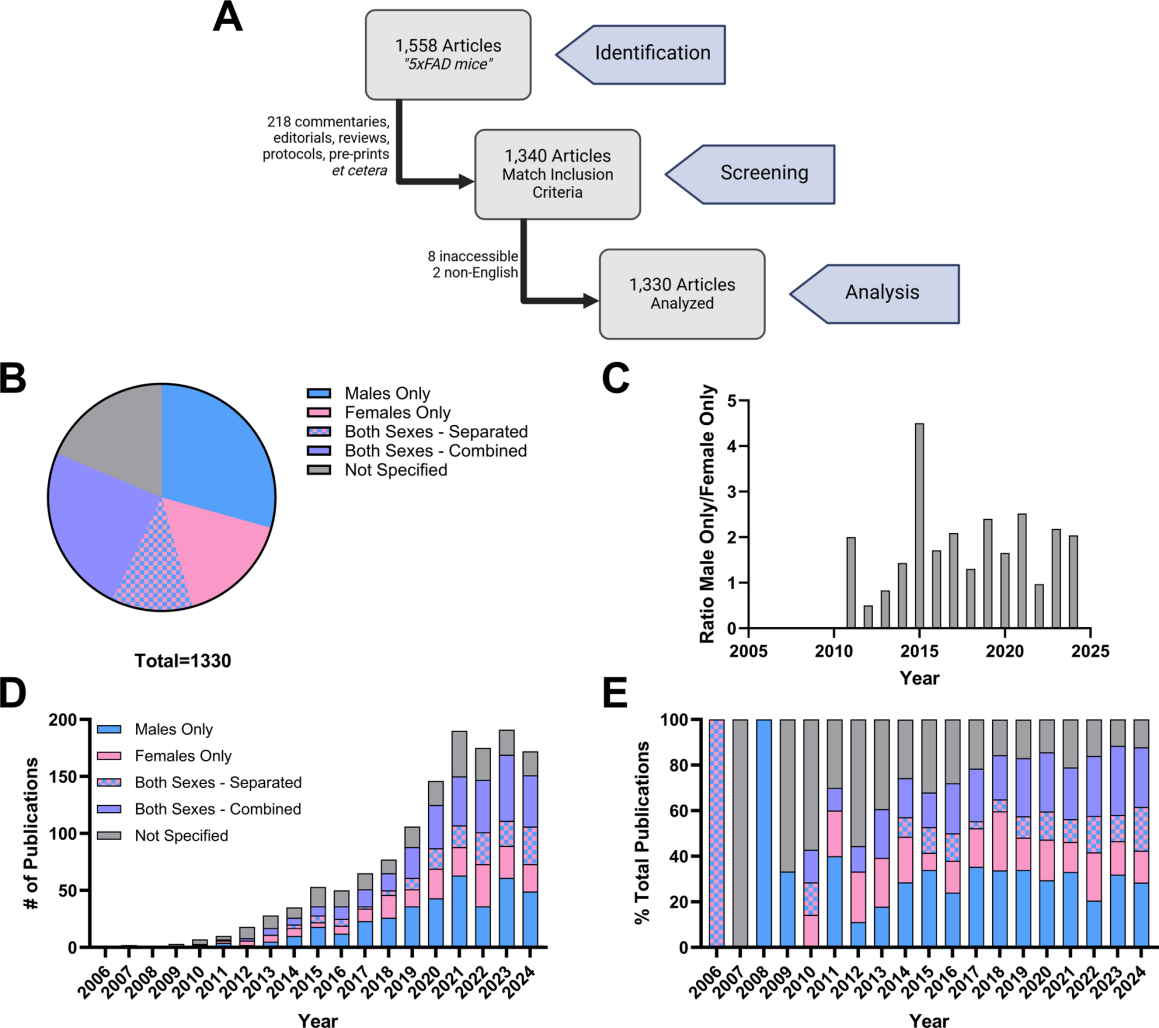
Analysis of subject sex in studies utilizing the 5xFAD model and published between 2006 and 2024. (**A**) Flow diagram depicting article screening. (**B**) Breakdown of reported sex of subjects for the entire history of the 5xFAD line. Χ^2^ = 126.7 indicating a significant deviation from expected (equal) distribution (*P* < 0.0001). (**C**) Ratio of manuscripts utilizing only male subjects to those using only female subjects as a function of year. The overall average was 1.8:1. Reported sex of 5xFAD subjects is also broken down by year either as (**D**) total manuscripts in each category or (**E**) a percent of the total number of papers on the 5xFAD line published that year. Two-way ANOVA revealed a significant effect of category [subject sex, F [4, 72] = 9.03; *P* < 0.0001] and year [F [18, 72] = 20.23; *P* < 0.0001] on the dataset in (**C**)

A search for “5xFAD mice” on PubMed returned a total of 1,558 articles published between 2006 (first report) and 2024 (as of January 21 st, 2025). We excluded non-research reports including commentaries, editorials, reviews, protocols/methods, and retracted articles from analysis. In addition, manuscripts wherein additional genetic modifications were introduced onto a 5xFAD background, but a non-transgenic control group was not included were removed, yielding 1,340 articles. Ten further papers were eliminated as we could not access the full text manuscript [8], or they were not written in English and no translated text was available [2]. Ultimately, a total of 1,330 articles were analyzed and scored (Fig. 2A).

For each article we coded the manuscripts based on the sex of 5xFAD mice utilized. Several manuscripts failed to mention subject sex and were coded as “sex not specified”. Articles coded “males only” or “females only” utilized exclusively a single sex. For a paper to be assigned to the “both sexes – separated” category, at least one dataset provided must have data independently tabulated for males and females with an analysis of potential sex effects provided. We included manuscripts in this category if data were shown for a single sex or combined in the main figures, but a sex-dependent analysis was provided in the data supplement. The final code, “both sexes – combined”, was assigned to manuscripts in which the authors noted that mice of both sexes were used, but no analysis of sex differences is provided. This category included papers where all datasets combined the two sexes in varying ratios; where some datasets were with one sex, but others with the opposite sex; and where data points are coded by sex, but the study was either underpowered to assess sex differences or data presentation precludes a sex-based analysis.

As we scored the manuscripts, we also made note of what, if any, justification was provided for selection of subject sex. The rationales provided fell into several categories: data were combined as no sex differences were found; data were combined despite noted sex differences; concerns regarding within- or between-sex variability; the accelerated, exacerbated, and/or reproducible pathology noted in female 5xFAD mice; the higher incidence of AD in women; precedent or to compare to prior work; limiting vertebrate animal use; and a few considerations specific to the 5xFAD model including the aggressiveness found in 5xFAD males and phenotypes found in only one sex. We also noted when authors acknowledged that a lack of consideration of sex as a biological variable was a limitation for their study.

### Statistical analysis 

Χ^2^ (Chi-square) tests were used to determine if observed group proportions significantly deviated from expected ratios (e.g., equal distribution among all groups). Two-way analysis of variance (ANOVA) was used to test for main effects in datasets containing proportions as a function of time. Statistical analyses were performed using GraphPad Prism software (v10).

## Results

### Male only or combined sex studies predominate articles published on the 5xFAD line

When considering the compendium of published knowledge on the 5xFAD mouse line (Fig. 2B), the largest proportion of articles used only male subjects (29.3%), followed by studies using both sexes but failing to account for potential sex differences within the datasets (24.2%). Indeed, manuscripts considering the modulatory influence of sex were in the minority (11.5%), behind articles where subject sex is not mentioned (18.6%) and those using females only (16.3%). It is worth noting that, whereas prior analysis of subject sex across neuroscience studies reported a ratio of male: female only reports at 5.5:1 [52], the ratio across studies of the 5xFAD line hovered around 2:1 with an overall average of 1.8:1 (Fig. 2C).

We next assessed whether subject sex representation changed over the history of the 5xFAD line (2006–2024). As we observed across AD mouse models (Fig. 1A), studies utilizing the 5xFAD line have increased overtime, peaking within the last 5 years at approximately 150 publications/year (Fig. 2D). When subject sex was expressed as a proportion of the total publications, a few trends emerged. First, with time, there has been a gradual decrease in manuscripts wherein subject sex is not specified and this category is replaced with studies utilizing both sexes either in combination or separately (Fig. 2E). In contrast, there was very little variation across the last decade (2014–2024) in studies of the 5xFAD line using only male or only female animals (Fig. 2E).

### A lack of sex differences observed is the most common rationale for omitting SABV analyses

**Fig. 3:**
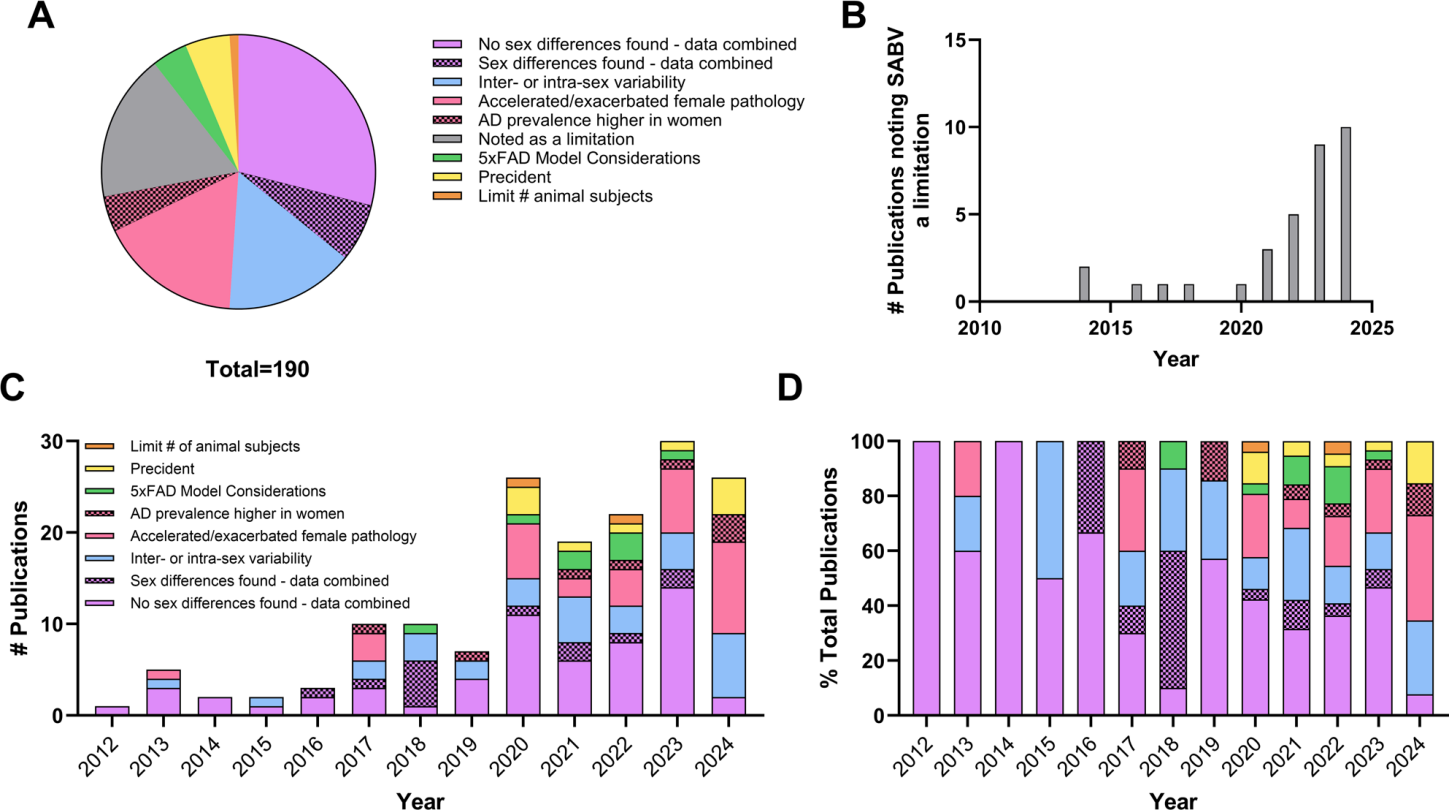
Tabulation of noted reasons for not separating data by sex in studies using the 5xFAD model published between 2012 and 2024. (**A**) Breakdown of rationales provided for excluding SABV over the history of the 5xFAD model. Χ^2^ = 111.5 indicating a significant deviation from the expected (equal) distribution (*P* < 0.0001). (**B**) Articles acknowledging that a lack of consideration of SABV is a study limitation throughout the history of the 5xFAD model. Justifications for not separating datasets by sex is broken down by year either as (**C**) total manuscripts in each rationale category or (**D**) as a percent of the total number of papers containing rationales published within that year. Two-way ANOVA revealed a significant effect of rationale [F [7, 84] = 7.282; *P* < 0.0001] and year [F [12, 84] = 3.745; *P* = 0.0002] on the dataset in (**C**)

We next tabulated rationales provided for not separating results based on sex of the subjects. Among the articles that provided justifications for lack of consideration of SABV (*n* = 190/1,330 or 14.3%), the largest proportion combined data because no sex differences were found (29.0%) followed by the accelerated pathology in female 5xFAD mice (16.8%) and concerns over inter-/intra-sex variability (15.2%), which included confounding influences of ovarian hormones in females or studies that noted the potential for differences between male and female subjects as a justification to use only one sex. (Fig. 3A). Of all the articles for the 5xFAD model, there was a total of 33 articles (17.4%) that noted that not considering SABV was a limitation of their study peaking at 10 articles in 2024 (Fig. 3B). The justification provided in the fewest number of articles included combination of data because sex differences are present (6.8%), precedence (e.g., males used in prior studies or a phenotype previously found in only one sex; 5.3%), the higher prevalence of AD in women (4.2%), specific considerations of the 5xFAD model including aggression in males and the collapsing of sex differences as a function of age (4.2%), and limitations in the number of subjects available (1.1%) (Fig. 3A). Next, we looked at breakdown of rationales for not separating datasets by sex and assessed whether the justifications had changed throughout the history of the 5xFAD line. Although the 5xFAD model has been around since 2006, we found no papers listing a rationale for choice of subject sex before 2012. Notably, the presence of text acknowledging the potential importance of SABV peaked within the past 5 years (Fig. 3C) at which point a broader array of rationales for the exclusion or inclusion of subject sex emerged (Fig. 3D).

### Artificial inflation of AD pathology in 5xFAD females due to the estrogen response element (ERE) in the Thy1 promotor driving transgene expression

**Fig. 4:**
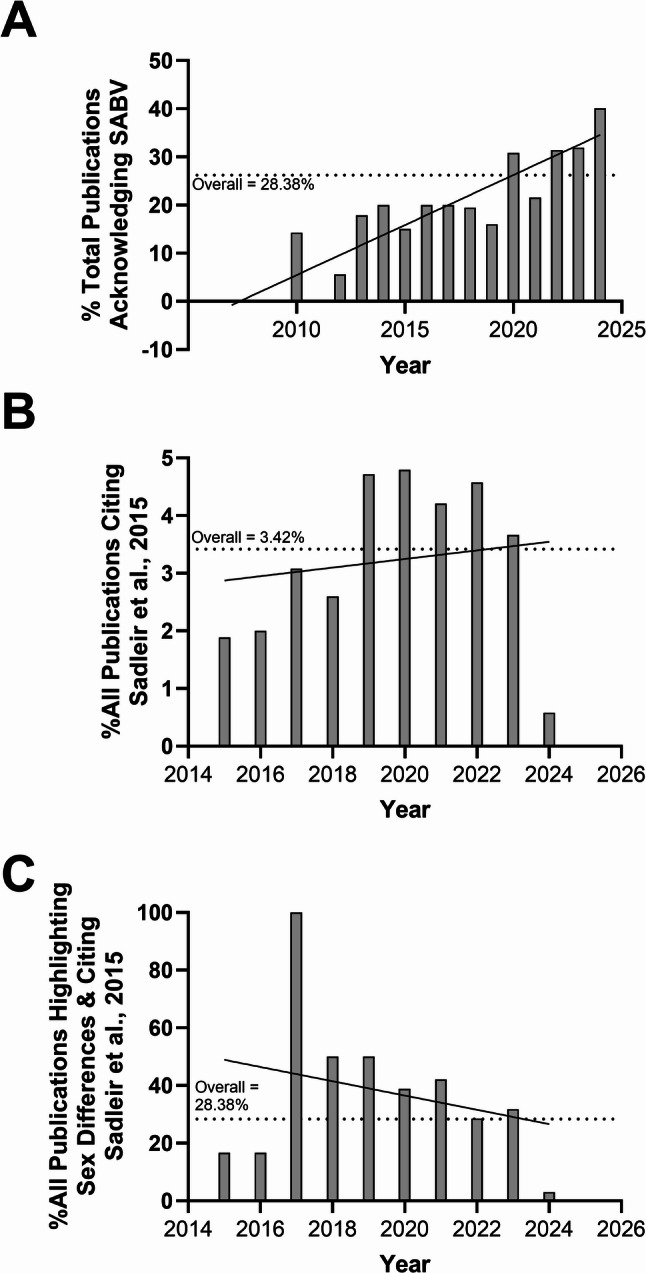
Acknowledgement of SABV in manuscripts published on the 5xFAD model overtime. (**A**) Percentage of total publications acknowledging SABV by year from 2007–2025. Simple linear regression revealed a significant increase with time [R-Squared = 0.834; *P* < 0.0001]. (**B**) Percentage of all publications by year from 2015–2024 citing the Sadleir et al., 2015 study. Simple linear regression revealed no significant change with time in number of publications citing Sadleir et al., 2015 [R-Squared = 0.025; *P* = 0.663]. (**C**) Percentage of all publications by year highlighting sex differences and citing Sadleir et al., 2015. Simple linear regression revealed no significant change with time [R-Squared = 0.079; *P* = 0.432]

Overall, there has been a linear increase (R^2^ = 0.834; *P* < 0.0001) in publications on the 5xFAD model that either separate datasets by sex, acknowledge that exclusion of SABV is a study limitation, or provide a rationale for using subjects of only one sex beginning around 2010 (Fig. 4A), a date that precedes implementation of the National Institutes of Health (NIH) mandate in January of 2016 requiring consideration of SABV in research design, analysis, and reporting for all submitted grant applications. Several studies have noted accelerated amyloid pathology in female 5xFAD mice relative to males and sex differences across a spectrum of physiological and behavioral phenotypes, the majority of which favor exacerbated impacts in females (Table 2). However, in 2015, Sadleir et al., published a key finding noting that 5xFAD females may exhibit higher levels of Aβ_42_ compared to male mice due to an estrogen response element (ERE) in the transgene *Thy1* promoter [58]. Of all papers published on the 5xFAD mouse, only 3.4% cite the Sadleir study (Fig. 4B). In addition, whereas a larger proportion of studies highlighting sex differences in the 5xFAD model mention the potential confounding effect of sex-biased transgene expression, less than a third (28.4%) of these studies cite Sadleir et al., 2015 (Fig. 4C). Notably, we found no trend in the rate of citation of the Sadleir study (R^2^ = 0.025; *P* = 0.663; Fig. 4B) or in studies looking at or acknowledging sex differences (R^2^ = 0.079; *P* = 0.432; Fig. 4C) with time.

**Table 2:** Sex differences in the 5xFAD mouse model

Phenotype		Sex bias	References
*Pathology*			
	Amyloid β (Aβ) accumulation & deposition	F > M	[67, 69–75]
	Stress-induced amyloid deposition	F > M	[76, 77]
	Sensitivity to APOE genotype	F > M	[78–82]
*Physiology*			
	Sleep-wake cycle fragmentation	F > M	[83]
	Cerebral amyloid angiopathy	F > M	[78]
	Impaired hippocampal neurogenesis	M > F	[84]
	Neuroinflammation & gliosis	F > M	[67, 74, 85–97]
	Cortical microbleeds	F > M	[80, 98]
	Altered metabolism and hepatic dysfunction	F > M	[99, 100]
	Bone Loss	M > F	[101]
*Behavior*			
	*Locomotor*		
	Altered horizontal locomotion	F > M	[67, 102, 103]
	Decreased rearing	F > M	[104]
	Impaired motor coordination on rotarod	F > M	[67, 104]
	*Sensory*		
	Altered whisker exploration	F > M	[105]
	Auditory gap detection deficits	M > F	[106]
	Altered odor detection/learning	F > M	[107, 108]
	*Anxiety & Repetitive*		
	Altered light/dark exploration	M > F	[109]
	Anxiety in elevated plus maze	F > M	[110–112]
	Altered nest building/burrowing	F > M	[113]
	*Cognitive*		
	Impaired working memory in Y maze	M > F	[75, 109, 110, 114, 115]
	Impaired maze learning & reversal learning	F > M	[66, 75, 103, 110, 116–119]
	Impaired object recognition memory	F > M	[86, 118]
	Impaired fear conditioning	M > F	[80, 120, 121]
	Aggression and social deficits	M > F	[122]

Phenotypes of the 5xFAD mouse model reported to display sex differences or sex bias. Corresponding references are provided

### Female-specific enhancement in transgene expression alone cannot account for all sex differences noted across AD mouse models

**Fig. 5:**
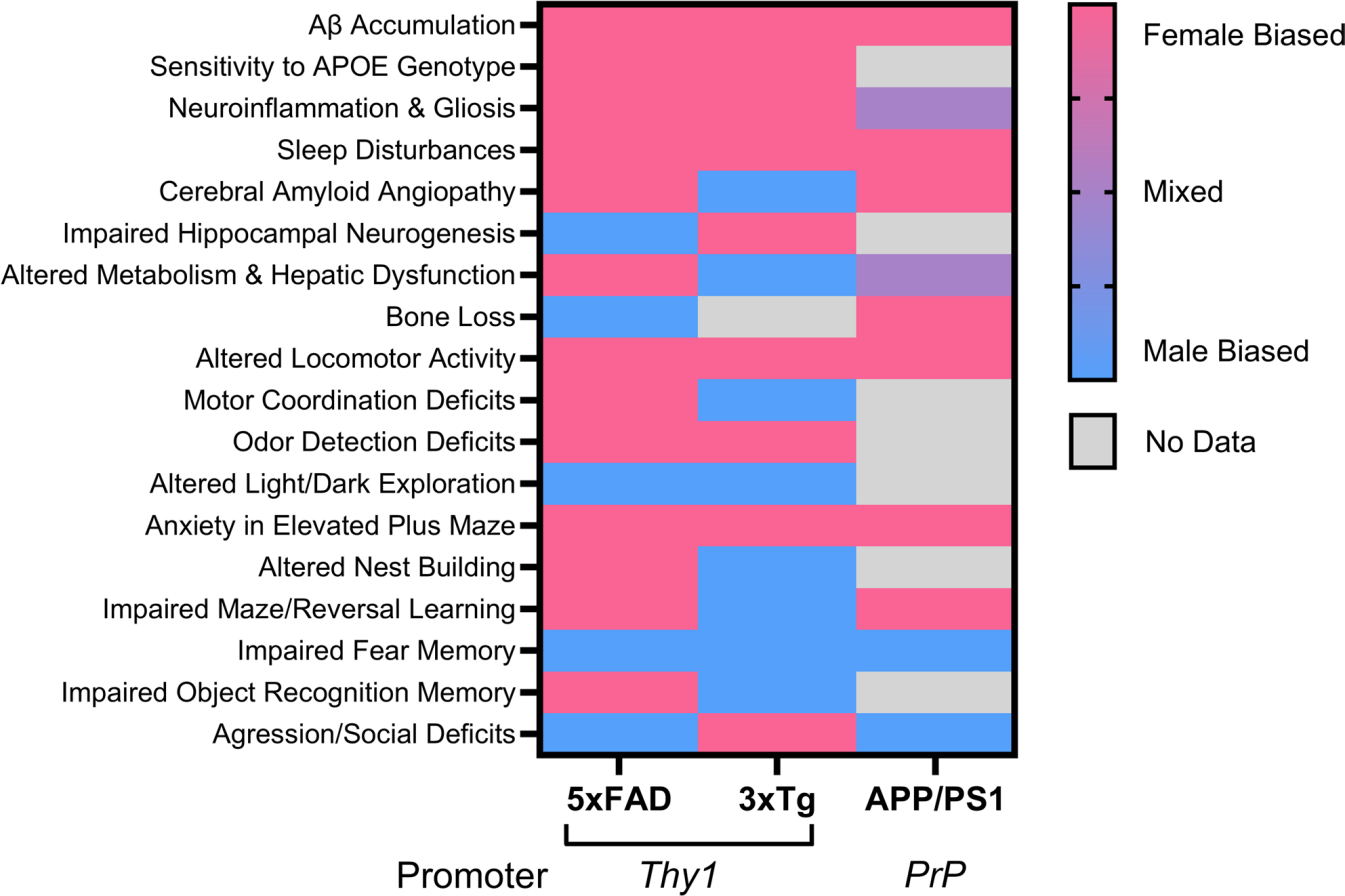
Sex-biased phenotypes of 3 AD mouse models. Pathological, physiological and behavioral phenotypes shared across the 5xFAD, 3xTg, and APP/PS1 mouse lines are depicted and coded based on whether they are noted to be male (blue)- or female (pink)-biased. For select endpoints, studies identifying both male- or female-specific impacts have been published and these are coded as “mixed” (purple)

If the primary factor driving female-biased pathology in the 5xFAD mouse model was the presence of the *Thy1* promoter ERE, sex differences in observed phenotypes in the line would be reflected in any AD model utilizing the *Thy1* promoter, but not a model for which transgene expression is driven by another promoter. However, when we surveyed sex differences noted in two additional AD mouse lines, the *Thy1* driven 3xTg (Table S1) and *PrP*-dependent APP/PS1 (Table S2), we noted several sex-biased alterations in pathological, physiological, and behavioral endpoints shared across all models (e.g., Aβ accumulation) (Fig. 5). In addition, there was a significant lack of congruency between the sex-bias in several phenotypes exhibited by the 5xFAD and 3xTg lines, despite both lines relying on the *Thy1* promoter to drive transgene expression (Fig. 5).

## Discussion

Despite clear sex biases in the rate of diagnosis and behavioral manifestations of AD, the potential biological underpinnings of these clinical observations remain largely unexplored. To determine the molecular basis for the prevalence of AD diagnoses in women over men, preclinical studies are required that utilize a face and construct valid disease model and evaluate endpoints in subjects of both sexes. As a collective, the field of neuroscience has historically been heavily skewed toward the exclusive study of male subjects [52], a bias that, as of 2019, had yet to change [53]. Here, we evaluated trends in subject sex for published studies utilizing the 5xFAD AD mouse model, one of the most widely disseminated transgenic mouse lines. Consistent with trends in the larger neuroscience field, most published reports on 5xFAD mice utilized only males, combined datasets from both sexes, or did not specify subject sex. Although the NIH requires grant applications submitted after January 2016 and carried out in vertebrate animals to factor SABV into research design, analyses, and reporting or provide a strong justification for single-sex investigations, the proportion of 5xFAD-related manuscripts reporting data separated by sex has not increased by a significant degree within the last decade. Rather, papers in which subject sex is not specified have largely been replaced by studies utilizing both sexes but combining data. However, it is important to note that, in most datasets combining data from both sexes, SABV was not factored into a priori study design, statistical analyses supporting a lack of sex differences were not provided, and, generally, experiments were underpowered to detect sex differences if they did exist. Indeed, prior analysis of articles claiming to identify phenotypic sex differences indicated these issues are particularly prevalent in the field of neuroscience [59]. We should note that, in 2024, the number of publications on the 5xFAD model that combined data from both sexes as “no sex differences were found” dropped precipitously (~ 7.5%) compared to the previous decade (~ 40%). We also found a consistent upward trend in the number of papers published that separate data by sex, provide a justification for the use of only one sex, or acknowledge the lack of sex differences analysis represents a study limitation beginning around 2010, though such publications still make up the minority (< 30%). Time will tell if this trend holds moving forward, but it could reflect recent pressure by scientific journals to acknowledge or include SABV, which appears to more effectively shift standard practice as compared with policies enacted at the funding level [56].

The claim that a single sex was chosen to either exclude between-sex variability or that males were chosen to eliminate the confounding influence of estrus cycling in females was common across manuscripts published on the 5xFAD line. Studies continue to use this justification despite recent evidence demonstrating that the long-standing assertion that cyclical hormone production leads to increased endpoint variability in female rodents is categorically false [54, 55]. This claim is particularly problematic with regards to hormone action in the brain where levels of steroid hormones can be several fold higher than circulating pools due to local production and processing [60–62] and do not necessarily correlate with plasma hormone content [63, 64]. The broadly held belief that estrogen and progestin action is more prevalent in the female brain is also flawed as in some brain regions, including the hippocampus, estradiol levels are higher in males due to aromatization of peripherally- and locally-derived androgens [61]. The perseverance of these prevailing presumptions, therefore, obfuscates the biological reality in which neurosteroid functions may be both shared or divergent between the sexes depending on the neurobiological context.

Given alterations in brain estradiol content have been found in AD patients [35], elucidating the neuroprotective impacts of estrogens is an area of active research. For example, drugs targeting both the membrane associated, G protein-coupled estrogen receptor (Gper1) and nuclear estrogen receptors have been tested for their impacts on amyloid pathology and cognitive function in the 5xFAD model [65, 66]. However, in our analysis of reported sex differences in the 5xFAD model, we identified evidence indicating that the design of the 5xFAD model introduced key pitfalls in interpretation of steroid hormone impacts in the 5xFAD brain. First, in 2015 Sadleir et al. noted that the *Thy1.2* promoter used to drive transgene expression in the 5xFAD line contains a putative estrogen response element (ERE), which they proposed may explain the more rapid development of plaque burden in 5xFAD females [58]. It is likely, therefore, that APP pathology in 5xFAD mice may not be solely shaped by endogenous mechanisms but also dependent on differential estrogen content across brain regions. For example, the higher estrogen content in the cortex of females vs. males may explain why amyloid plaque deposition is faster in cortical regions in 5xFAD females relative to other brain regions and patterns observed in males [67]. More recently, Sasmita and colleagues noted that parental origin also influences transgene expression in 5xFAD mice due to maternal imprinting in the *Thy1* promoter [68], an observation that may explain inter-study variability in phenotypic outcomes as breeding schemes are rarely reported. It is important to note, however, that the Thy1 promoter ERE alone cannot account for sex differences observed in 5xFAD mice. Indeed, though both the 5xFAD and 3xTg AD models utilize the Thy1 promoter to drive transgene expression, the lack of convergence in sex-biased phenotypes between lines (Fig. 5) indicates the existence of other neurobiological mechanisms shaping the sex-dependent impacts of pathogenic *APP*, *PS1*, and *Tau* mutants. Conversely, endpoints for which sex-bias persists across lines, including female-specific acceleration of amyloid deposition, sleep disruptions, altered locomotion, and anxiety and male-specific impairments in fear memory, may reflect phenotypes for which the modulatory influence of sex is particularly penetrant.

The potential confounds intrinsic to the 5xFAD model have not stymied the popularity of the line, which accounted for ~ 40% of papers on transgenic AD models in 2024. Indeed, awareness of these issues is limited as fewer than 4% of studies published after 2015 cite the Sadlier article, though this rate appears to be higher (~ 28%) for papers exploring sex differences in the 5xFAD model. Regardless, those seeking to use 5xFAD mice as a preclinical model of AD should be aware that it may be difficult to disentangle legitimate sex differences driven by genetic/gonadal sex from those predominantly arising from model design. Difficulty in elucidating the mechanistic bases for sex differences in the 5xFAD and other AD models has also likely been exacerbated due to a lack of consistent and systematic consideration of SABV. The development of novel AD therapeutics able to translate from pre-clinical studies to the clinic will require improvements to both the generation and implementation of rodent disease models, as well as deliberate attention to experimental design that incorporates the influence of biological sex on study endpoints.

## Supplementary Information

Supplementary Material 1

## Data Availability

The datasets used and/or analyzed during the current study are available from the corresponding author on reasonable request.
