# Supplementary material for: Consideration of sex as a biological variable over the history of the 5xFAD Alzheimer’s Disease mouse model
Source: Biol Sex Differ. 2025 Dec 17;16:105. doi: 10.1186/s13293-025-00788-3 (PMC12709748; doi:10.1186/s13293-025-00788-3)
Supplement: Supplementary file 1 — Supplementary Material 1 [file 13293_2025_788_MOESM1_ESM.docx]

| **Phenotype** | | **Sex bias** | **References** |
| --- | --- | --- | --- |
| *Pathology* |  |  |  |
|  | Amyloid β (Aβ) accumulation & deposition | F > M | (1-9) |
|  | Sensitivity to APOE genotype | F > M | (3, 10) |
|  |  |  |  |
| *Physiology* |  |  |  |
|  | Sleep-wake cycle fragmentation | F > M | (11) |
|  | Cerebral amyloid angiopathy | M > F | (12) |
|  | Impaired hippocampal neurogenesis | F > M | (1, 5, 12) |
|  | Neuroinflammation & gliosis | F > M | (1, 2, 13) |
|  | Altered metabolism and hepatic dysfunction | M > F | (3, 8, 14-16) |
|  | Adrenal Gland Hypertrophy | F > M | (17) |
|  |  |  |  |
| *Behavior* |  |  |  |
|  | *Locomotor* |  |  |
|  | Altered horizontal locomotion | F > M | (17, 18) |
|  | Decreased rearing | M > F | (10, 12, 18) |
|  | Impaired motor coordination on rotarod | M > F | (18-20) |
|  |  |  |  |
|  | *Sensory* |  |  |
|  | Altered odor detection/learning | F > M | (21) |
|  |  |  |  |
|  | *Anxiety & Repetitive* |  |  |
|  | Altered light/dark exploration | M > F | (19) |
|  | Anxiety in open field tests | F > M | (22, 23) |
|  | Anxiety in elevated plus maze | F > M | (24, 25) |
|  | Altered nest building/burrowing | M > F | (23, 26) |
|  | Anxiety in Hebb-Williams Maze | F > M | (25) |
|  |  |  |  |
|  | *Cognitive* |  |  |
|  | Impaired maze learning & reversal learning | M > F | (15, 27) |
|  | Impaired object recognition memory | M > F | (28, 29) |
|  | Impaired fear conditioning | M > F | (27, 30) |
|  | Aggression and social deficits | F > M | (23, 31) |
|  | Barnes Maze latency | F > M | (27) |

**Supplementary Table 1** - Phenotypes of the **3xTg** Alzheimer’s Disease (AD) mouse model reported to display sex differences or sex bias. Corresponding references are provided.

**Supplementary Table 2** - Phenotypes of the **APP/PS1** Alzheimer’s Disease (AD) mouse model reported to display sex differences or sex bias. Corresponding references are provided.

| **Phenotype** | | **Sex bias** | **References** |
| --- | --- | --- | --- |
| *Pathology* |  |  |  |
|  | Amyloid β (Aβ) accumulation & deposition | F > M | (32-36) |
|  | Shortened lifespan | F > M | (34, 37) |
|  |  |  |  |
| *Physiology* |  |  |  |
|  | Circadian rhythm disturbances | F > M | (38) |
|  | Astrocytosis, & gliosis | F > M | (39) |
|  | Plaque associated microglia | M > F | (40) |
|  | Neuroinflammation | M > F | (36) |
|  | Cerebral amyloid angiopathy and microhemorrhage | F > M | (39) |
|  | Altered metabolism and hepatic dysfunction | F > M | (41, 42) |
|  | Impaired glucose and insulin tolerance | M > F | (33, 35) |
|  | Elevated cholesterol & triglycerides | M > F | (33) |
|  | Periodontal bone loss | F > M | (43) |
|  | Elevated food consumption | M > F | (34) |
|  | Lower cerebral blood flow | F > M | (36) |
|  | Increased cortical thickness & hippocampal volume | F > M | (36) |
|  | Diminished modulatory hippocampal input | M > F | (44) |
|  |  |  |  |
| *Behavior* |  |  |  |
|  | *Locomotor* |  |  |
|  | Altered horizontal locomotion | F > M | (36) |
|  |  |  |  |
|  | *Anxiety & Repetitive* |  |  |
|  | Anxiety in elevated plus maze | F > M | (45) |
|  |  |  |  |
|  | *Cognitive* |  |  |
|  | Impaired spatial memory | F > M | (33, 46, 47) |
|  | Impaired Morris Water Maze performance | F > M | (35, 46, 47) |
|  | Impaired contextual fear memory | M > F | (48, 49) |
|  | Impaired associative memory | F > M | (50) |
|  | Aggression and social deficits | M > F | (34) |

**References**

1. Belfiore R, Rodin A, Ferreira E, Velazquez R, Branca C, Caccamo A, Oddo S. Temporal and regional progression of Alzheimer's disease-like pathology in 3xTg-AD mice. Aging Cell. 2019;18(1):e12873.

2. Jiao JJ, Hu Y, Cui YJ, Tuo CM, Wang YX, Li XY, et al. Anisomycin alleviates cognitive impairments and pathological features in 3xTg-AD mice. Neuropharmacology. 2024;261:110159.

3. Barber AJ, Del Genio CL, Swain AB, Pizzi EM, Watson SC, Tapiavala VN, et al. Age, sex and Alzheimer's disease: a longitudinal study of 3xTg-AD mice reveals sex-specific disease trajectories and inflammatory responses mirrored in postmortem brains from Alzheimer's patients. Alzheimers Res Ther. 2024;16(1):134.

4. Arsenault D, Tremblay C, Emond V, Calon F. Sex-dependent alterations in the physiology of entorhinal cortex neurons in old heterozygous 3xTg-AD mice. Biol Sex Differ. 2020;11(1):63.

5. Carroll JC, Rosario ER, Kreimer S, Villamagna A, Gentzschein E, Stanczyk FZ, Pike CJ. Sex differences in beta-amyloid accumulation in 3xTg-AD mice: role of neonatal sex steroid hormone exposure. Brain Res. 2010;1366:233-45.

6. Perez SE, He B, Muhammad N, Oh KJ, Fahnestock M, Ikonomovic MD, Mufson EJ. Cholinotrophic basal forebrain system alterations in 3xTg-AD transgenic mice. Neurobiol Dis. 2011;41(2):338-52.

7. Mendell AL, Creighton SD, Wilson HA, Jardine KH, Isaacs L, Winters BD, MacLusky NJ. Inhibition of 5alpha Reductase Impairs Cognitive Performance, Alters Dendritic Morphology and Increases Tau Phosphorylation in the Hippocampus of Male 3xTg-AD Mice. Neuroscience. 2020;429:185-202.

8. Nava Catorce M, Acero G, Gevorkian G. Age- and sex-dependent alterations in the peripheral immune system in the 3xTg-AD mouse model of Alzheimer's disease: Increased proportion of CD3+CD4-CD8- double-negative T cells in the blood. J Neuroimmunol. 2021;360:577720.

9. Vandal M, White PJ, Chevrier G, Tremblay C, St-Amour I, Planel E, et al. Age-dependent impairment of glucose tolerance in the 3xTg-AD mouse model of Alzheimer's disease. FASEB J. 2015;29(10):4273-84.

10. Canete T, Gimenez-Llort L. Preserved Thermal Pain in 3xTg-AD Mice With Increased Sensory-Discriminative Pain Sensitivity in Females but Affective-Emotional Dimension in Males as Early Sex-Specific AD-Phenotype Biomarkers. Front Aging Neurosci. 2021;13:683412.

11. Stover KR, Campbell MA, Van Winssen CM, Brown RE. Analysis of motor function in 6-month-old male and female 3xTg-AD mice. Behav Brain Res. 2015;281:16-23.

12. Muntsant A, Jimenez-Altayo F, Puertas-Umbert L, Jimenez-Xarrie E, Vila E, Gimenez-Llort L. Sex-Dependent End-of-Life Mental and Vascular Scenarios for Compensatory Mechanisms in Mice with Normal and AD-Neurodegenerative Aging. Biomedicines. 2021;9(2).

13. Gannon OJ, Robison LS, Salinero AE, Abi-Ghanem C, Mansour FM, Kelly RD, et al. High-fat diet exacerbates cognitive decline in mouse models of Alzheimer's disease and mixed dementia in a sex-dependent manner. J Neuroinflammation. 2022;19(1):110.

14. Kapadia M, Mian MF, Michalski B, Azam AB, Ma D, Salwierz P, et al. Sex-Dependent Differences in Spontaneous Autoimmunity in Adult 3xTg-AD Mice. J Alzheimers Dis. 2018;63(3):1191-205.

15. Kapadia M, Mian MF, Ma D, Hutton CP, Azam A, Narkaj K, et al. Differential effects of chronic immunosuppression on behavioral, epigenetic, and Alzheimer's disease-associated markers in 3xTg-AD mice. Alzheimers Res Ther. 2021;13(1):30.

16. Fraile-Ramos J, Reig-Vilallonga J, Gimenez-Llort L. Glomerular Hypertrophy and Splenic Red Pulp Degeneration Concurrent with Oxidative Stress in 3xTg-AD Mice Model for Alzheimer's Disease and Its Exacerbation with Sex and Social Isolation. Int J Mol Sci. 2024;25(11).

17. Baeta-Corral R, De la Fuente M, Gimenez-Llort L. Sex-dependent worsening of NMDA-induced responses, anxiety, hypercortisolemia, and organometry of early peripheral immunoendocrine impairment in adult 3xTg-AD mice and their long-lasting ontogenic modulation by neonatal handling. Behav Brain Res. 2023;438:114189.

18. Castillo-Mariqueo L, Gimenez-Llort L. Impact of Behavioral Assessment and Re-Test as Functional Trainings That Modify Survival, Anxiety and Functional Profile (Physical Endurance and Motor Learning) of Old Male and Female 3xTg-AD Mice and NTg Mice with Normal Aging. Biomedicines. 2022;10(5).

19. Alveal-Mellado D, Castillo-Mariqueo L, Gimenez-Llort L. Sex- and Neuropsychiatric-Dependent Circadian Alterations in Daily Voluntary Physical Activity Engagement and Patterns in Aged 3xTg-AD Mice. Int J Mol Sci. 2022;23(22).

20. Garvock-de Montbrun T, Fertan E, Stover K, Brown RE. Motor deficits in 16-month-old male and female 3xTg-AD mice. Behav Brain Res. 2019;356:305-13.

21. Gur E, Fertan E, Kosel F, Wong AA, Balci F, Brown RE. Sex differences in the timing behavior performance of 3xTg-AD and wild-type mice in the peak interval procedure. Behav Brain Res. 2019;360:235-43.

22. Jimenez-Altayo F, Sanchez-Ventura J, Vila E, Gimenez-Llort L. Crosstalk between Peripheral Small Vessel Properties and Anxious-like Profiles: Sex, Genotype, and Interaction Effects in Mice with Normal Aging and 3xTg-AD mice at Advanced Stages of Disease. J Alzheimers Dis. 2018;62(4):1531-8.

23. Torres-Lista V, Gimenez-Llort L. Vibrating Tail, Digging, Body/Face Interaction, and Lack of Barbering: Sex-Dependent Behavioral Signatures of Social Dysfunction in 3xTg-AD Mice as Compared to Mice with Normal Aging. J Alzheimers Dis. 2019;69(4):969-77.

24. Fertan E, Stover KRJ, Brant MG, Stafford PM, Kelly B, Diez-Cecilia E, et al. Effects of the Novel IDO Inhibitor DWG-1036 on the Behavior of Male and Female 3xTg-AD Mice. Front Pharmacol. 2019;10:1044.

25. Fertan E, Wong AA, Vienneau NA, Brown RE. Age and sex differences in motivation and spatial working memory in 3xTg-AD mice in the Hebb-Williams maze. Behav Brain Res. 2019;370:111937.

26. Torres-Lista V, Gimenez-Llort L. Impairment of nesting behaviour in 3xTg-AD mice. Behav Brain Res. 2013;247:153-7.

27. Stover KR, Campbell MA, Van Winssen CM, Brown RE. Early detection of cognitive deficits in the 3xTg-AD mouse model of Alzheimer's disease. Behav Brain Res. 2015;289:29-38.

28. Duggan MR, Steinberg Z, Peterson T, Francois TJ, Parikh V. Cognitive trajectories in longitudinally trained 3xTg-AD mice. Physiol Behav. 2024;275:114435.

29. Clinton LK, Billings LM, Green KN, Caccamo A, Ngo J, Oddo S, et al. Age-dependent sexual dimorphism in cognition and stress response in the 3xTg-AD mice. Neurobiol Dis. 2007;28(1):76-82.

30. Pairojana T, Phasuk S, Suresh P, Huang SP, Pakaprot N, Chompoopong S, et al. Age and gender differences for the behavioral phenotypes of 3xTg alzheimer's disease mice. Brain Res. 2021;1762:147437.

31. Nguyen ET, Selmanovic D, Maltry M, Morano R, Franco-Villanueva A, Estrada CM, Solomon MB. Endocrine stress responsivity and social memory in 3xTg-AD female and male mice: A tale of two experiments. Horm Behav. 2020;126:104852.

32. Wang J, Tanila H, Puolivali J, Kadish I, van Groen T. Gender differences in the amount and deposition of amyloidbeta in APPswe and PS1 double transgenic mice. Neurobiol Dis. 2003;14(3):318-27.

33. Li X, Feng Y, Wu W, Zhao J, Fu C, Li Y, et al. Sex differences between APPswePS1dE9 mice in A-beta accumulation and pancreatic islet function during the development of Alzheimer's disease. Lab Anim. 2016;50(4):275-85.

34. Pugh PL, Richardson JC, Bate ST, Upton N, Sunter D. Non-cognitive behaviours in an APP/PS1 transgenic model of Alzheimer's disease. Behav Brain Res. 2007;178(1):18-28.

35. Mifflin MA, Winslow W, Surendra L, Tallino S, Vural A, Velazquez R. Sex differences in the IntelliCage and the Morris water maze in the APP/PS1 mouse model of amyloidosis. Neurobiol Aging. 2021;101:130-40.

36. Lohkamp KJ, Timmer N, Sole Guardia G, Shenk J, Verweij V, Geenen B, et al. Sex-Specific Adaptations in Alzheimer's Disease and Ischemic Stroke: A Longitudinal Study in Male and Female APP(swe)/PS1(dE9) Mice. Life (Basel). 2025;15(3).

37. Roberts H, Fang Y, Quinn K, Hill T, Peck MR, Bartke A, et al. Lifespan of male and female APP/PS1 and APP(NL-F/NL-F) mouse models of Alzheimer's disease. J Alzheimers Dis. 2025;105(1):56-61.

38. Carrero L, Antequera D, Alcalde I, Megias D, Ordonez-Gutierrez L, Gutierrez C, et al. Altered Clock Gene Expression in Female APP/PS1 Mice and Aquaporin-Dependent Amyloid Accumulation in the Retina. Int J Mol Sci. 2023;24(21).

39. Jiao SS, Bu XL, Liu YH, Zhu C, Wang QH, Shen LL, et al. Sex Dimorphism Profile of Alzheimer's Disease-Type Pathologies in an APP/PS1 Mouse Model. Neurotox Res. 2016;29(2):256-66.

40. Pait MC, Kaye SD, Su Y, Kumar A, Singh S, Gironda SC, et al. Novel method for collecting hippocampal interstitial fluid extracellular vesicles (EV(ISF) ) reveals sex-dependent changes in microglial EV proteome in response to Abeta pathology. J Extracell Vesicles. 2024;13(1):e12398.

41. Trushina E, Nemutlu E, Zhang S, Christensen T, Camp J, Mesa J, et al. Defects in mitochondrial dynamics and metabolomic signatures of evolving energetic stress in mouse models of familial Alzheimer's disease. PLoS One. 2012;7(2):e32737.

42. Wu J, Fu B, Lei H, Tang H, Wang Y. Gender differences of peripheral plasma and liver metabolic profiling in APP/PS1 transgenic AD mice. Neuroscience. 2016;332:160-9.

43. Chen H, Liao Y, Zhang X, Shen H, Shang D, He Z, et al. Age- and sex-related differences of periodontal bone resorption, cognitive function, and immune state in APP/PS1 murine model of Alzheimer's disease. J Neuroinflammation. 2023;20(1):153.

44. Luo H, Marron Fernandez de Velasco E, Gansemer B, Frederick M, Aguado C, Lujan R, et al. Amyloid-beta oligomers trigger sex-dependent inhibition of GIRK channel activity in hippocampal neurons in mice. Sci Signal. 2024;17(856):eado4132.

45. Cuervo-Zanatta D, Garcia-Mena J, Perez-Cruz C. Gut Microbiota Alterations and Cognitive Impairment Are Sexually Dissociated in a Transgenic Mice Model of Alzheimer's Disease. J Alzheimers Dis. 2021;82(s1):S195-S214.

46. Gallagher JJ, Minogue AM, Lynch MA. Impaired performance of female APP/PS1 mice in the Morris water maze is coupled with increased Abeta accumulation and microglial activation. Neurodegener Dis. 2013;11(1):33-41.

47. Britz J, Ojo E, Dhukhwa A, Saito T, Saido TC, Hascup ER, et al. Assessing Sex-Specific Circadian, Metabolic, and Cognitive Phenotypes in the AbetaPP/PS1 and APPNL-F/NL-F Models of Alzheimer's Disease. J Alzheimers Dis. 2022;85(3):1077-93.

48. Kommaddi RP, Verma A, Muniz-Terrera G, Tiwari V, Chithanathan K, Diwakar L, et al. Sex difference in evolution of cognitive decline: studies on mouse model and the Dominantly Inherited Alzheimer Network cohort. Transl Psychiatry. 2023;13(1):123.

49. Simon ZD, McFarland KN, Golde TE, Chakrabarty P, Febo M. Sex dependent effect of amyloidosis on functional network 'hub' topology is associated with downregulated neuronal gene signatures in the APP swe /PSEN1dE9 double transgenic mouse. bioRxiv. 2025.

50. Navakkode S, Gaunt JR, Pavon MV, Bansal VA, Abraham RP, Chong YS, et al. Sex-specific accelerated decay in time/activity-dependent plasticity and associative memory in an animal model of Alzheimer's disease. Aging Cell. 2021;20(12):e13502.
